# From Webinars to Impact: Reenvisioning Clinical Guideline Ecosystem in Africa

**DOI:** 10.1002/gin2.70059

**Published:** 2026-01-16

**Authors:** Akah Thelma Eni, Ernest Alang Wung, Awah Noella, Ngeh Etienne, Karen DiValerio Gibbs, Guideline International Network‐ Africa Regional Community, Ebunoluwa Ayinmode

**Affiliations:** ^1^ Guidelines International Network, Pitlochry Scotland UK; ^2^ Effective Basic Services (eBASE) Africa Bamenda Cameroon; ^3^ Department of Public Health, Faculty of Health Sciences University of Buea, Molyko Buea Southwest Cameroon; ^4^ Department of Economics Policy Analysis, Faculty of Economics and Management University of Dschang Dschang Cameroon; ^5^ The University of Salford, The Crescent, Salford Greater Manchester UK; ^6^ The University of Texas Health Science Center at Houston: Houston Texas USA; ^7^ West African Institute for Applied Health Research (WAFERs) Ibadan Oyo State Nigeria

**Keywords:** AI, digital capacity‐building, GIN Africa, guidelines implementation

Guidelines are like a compass for putting evidence into practice in global health. They serve as a blueprint for quality care, decision‐making guidelines, and, when executed correctly, a lifeline for equitable health outcomes [1]. However, across low and middle‐income countries (LMICS), particularly Africa, it is challenging to transition from developing guidelines to implementing them [2]. Imported guidelines often overlook the nuanced details of local context [3], and systemic barriers, such as limited capacity and weak infrastructure, hinder their implementation. Against this backdrop, the Guidelines International Network (GIN) Africa regional community launched a daring initiative: could a simple webinar series spark a paradigm shift on how guidelines are developed, shared, and implemented across the continent [4]? The outcome was not merely affirmative but sparked scholarly reflections.

Between 2024 and 2025, GIN Africa hosted a series of webinars that went beyond the traditional ‘talkshop’ model. While individual presentations provided context and case examples, the ideas in this editorial reflect recurring themes that emerged across the discussions, regardless of the speaker or specific topic. These sessions became living repositories where new ideas like the Practical Approach to Care Kit (PACK) [5] and AI‐powered tools like AISHA have shown how locally designed, scalable solutions can transform the way clinical guidelines come to life. However, this movement encompasses more than just digital tools and frameworks. Rethinking guideline use begins with inclusivity, context, and local ownership. This editorial invites scholarly and professional reflection on how webinars shape knowledge, behavior, and guideline practices in LMICS. Four consistent priorities emerged from the discussions: building capacity, strengthening partnerships, using innovation wisely, and broadening engagement in guidelines.

Effective guideline development cannot happen in isolation. It depends on strong cross‐regional/transnational partnerships that enable knowledge exchange, alignment of experiences, and the creation of recommendations that are both globally robust and locally relevant. Such partnerships foster resource sharing, build capacity, and harmonize standards, factors that improve the adoption of guidelines across diverse settings. Also, transnational collaboration encourages cross‐system learning, while cross‐disciplinary engagement ensures recommendations are both evidence‐based and practical. Equally important is cross‐sectoral involvement, bringing together government, civil society, the private sector, and communities. This broad participation enhances credibility, strengthens relevance, and builds shared ownership, all of which support sustainable implementation.

Capacity building is especially critical in LMICs, where resources are often limited. Initiatives such as the *International Guideline Development & Evaluation Program (INGUIDE)* and the *Grading of Recommendations Assessment, Development and Evaluation (GRADE)* framework provide standardized tools. In sub‐Saharan Africa, for example, GRADE workshops have supported the creation of malaria and maternal health guidelines that align with global evidence while reflecting local contexts. These efforts equip multidisciplinary teams with the skills to produce practical, evidence‐based recommendations tailored to the realities of their settings. Ultimately, sustained capacity building is what enables LMICs to move from reliance on external guidance to producing, adapting, and implementing high‐quality guidelines that reflect local realities.

Innovation plays a critical role in improving the relevance, accessibility, and impact of guidelines. Digital platforms, living guidelines, and rapid adaptation approaches allow guidance to remain current, while the responsible use of artificial intelligence can support faster evidence synthesis and more context‐sensitive recommendations. Innovation also extends beyond technology to how guidance is communicated and used clear, accessible formats and inclusive engagement with patients, caregivers, and end‐users strengthen trust, equity, and real‐world implementation. Involving early‐career professionals further sustains innovation by building skills, leadership, and long‐term capacity, ensuring that evidence‐based guidance remains relevant, adaptable, and future‐ready.

Expanding guideline engagement beyond traditional health stakeholders increases societal relevance and encourages multisectoral adoption. In Africa, raising awareness and building capacity for evidence‐based guideline use has improved clinical practice, maternal and child health, and health system efficiency. Combining education, community engagement, and professional development helps translate scientific evidence into practical actions that meet local health needs. The GIN Africa webinar series reframed guidelines as locally driven, context‐sensitive tools, highlighting African innovations like PACK and AISHA and promoting cross‐sector collaboration. Its legacy lies in turning insights into investments, scaling homegrown models, and embedding inclusive guidelines into routine care, positioning Africa as a leader in translating evidence into practice globally.

GIN Africa will continue the webinar series with a clear shift toward facilitated dialogue, shared problem‐solving, and the co‐creation of adaptable resources, alongside stronger regional mentorship and South–South exchange. For GIN groups across high‐income and LMIC settings, the central lesson is that webinars drive real progress when they move beyond presentations to enable honest exchange and collective action.

## Author Contributions

**Akah Thelma Eni:** conceptualization of the editorial topic, overall supervision and critical revision of the manuscript. **Ernest Alang Wung:** methodological perspective, critical intellectual input, and manuscript revision. **Awah Noella:** methodological perspective, critical intellectual input, and manuscript revision. **Ngeh Etienne:** methodological perspective, critical intellectual input, and manuscript revision. **Karen DiValerio Gibbs:** supervision, conceptualization. Guideline International Network – Africa Regional Community – Final approval of the version to be published. **Ebunoluwa Ayinmode:** writing – original draft, validation, supervision, writing – review and editing, project administration.

## Funding

The authors received no specific funding for this work.

## Conflicts of Interest

The authors declare no conflicts of interest.

## Data Availability

Data sharing is not applicable to this article, as no new data were created or analyzed in this study.
